# A balance between membrane elasticity and polymerization energy sets the shape of spherical clathrin coats

**DOI:** 10.1038/ncomms7249

**Published:** 2015-02-19

**Authors:** Mohammed Saleem, Sandrine Morlot, Annika Hohendahl, John Manzi, Martin Lenz, Aurélien Roux

**Affiliations:** 1 Department of Biochemistry, University of Geneva, CH-1211 Geneva, Switzerland; 2 PCC, UMR168, Institut Curie/CNRS/Université Pierre et Marie Curie, F-75248 Paris, France; 3 University LPTMS, UMR 8626, CNRS/Université Paris Sud, Orsay F-91405, France; 4 Swiss National Centre for Competence in Research Programme Chemical Biology, CH-1211 Geneva, Switzerland

## Abstract

In endocytosis, scaffolding is one of the mechanisms to create membrane curvature by moulding the membrane into the spherical shape of the clathrin cage. However, the impact of membrane elastic parameters on the assembly and shape of clathrin lattices has never been experimentally evaluated. Here, we show that membrane tension opposes clathrin polymerization. We reconstitute clathrin budding *in vitro* with giant unilamellar vesicles (GUVs), purified adaptors and clathrin. By changing the osmotic conditions, we find that clathrin coats cause extensive budding of GUVs under low membrane tension while polymerizing into shallow pits under moderate tension. High tension fully inhibits polymerization. Theoretically, we predict the tension values for which transitions between different clathrin coat shapes occur. We measure the changes in membrane tension during clathrin polymerization, and use our theoretical framework to estimate the polymerization energy from these data. Our results show that membrane tension controls clathrin-mediated budding by varying the membrane budding energy.

## Abstract (web summary)

A relationship between membrane tension and clathrin polymerization during endocytosis has not been experimentally established. Here, the authors show using an *in vitro* reconstituted system and theoretical modelling that membrane tension regulates clathrin polymerization into spherical cages by varying the membrane budding energy.

## Graphical abstract

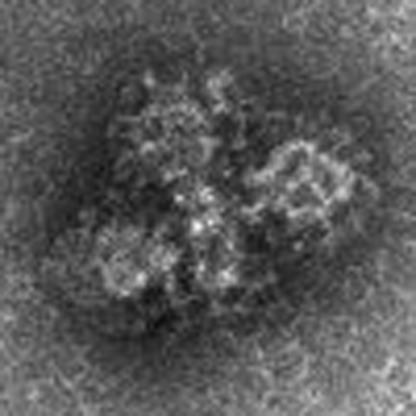

Budding of spherical vesicles is essential for membrane trafficking. Clathrin was the first protein found to be involved in this process^1^,^2^. Clathrin polymerizes into truncated icosahedral cages *in vitro*^3^ and together with various partners^4^ deforms the membrane into a spherical bud^5^,^6^,^7^. Among the mechanisms involved in bud formation, amphipathic insertions, which act as wedges to create curvature, and the crowding effect^8^ of proteins binding asymmetrically to the membrane have been investigated. On the contrary, clathrin was proposed to act by scaffolding, meaning that a protein coat of a given shape forces the membrane to curve^4^. The structure of the smallest clathrin cage is known in great detail^9^,^10^,^11^. However, clathrin also forms flat hexagonal lattices at the plasma membrane of cells^12^, and spherical coated vesicles of various sizes, ranging from 35 to 200 nm, in various organisms^13^. This variability in size and shape has called into question the initially proposed membrane scaffolding mechanism based on clathrin polymerization.

Clathrin binds membranes through a wide variety of adaptors including adaptin protein complexes (AP-1, AP-2, AP-3 and AP-4), AP180/CALM, GGAs and Hrs^14^. Adaptors are specific to subsets of clathrin-mediated membrane traffic pathways. In particular, AP180 has a high ability to polymerize clathrin *in vitro*, and forms smaller clathrin cages than clathrin alone^15^. AP180 has an ANTH domain that binds phosphatidylinositol 4,5-bisphosphate (PIP_2_)^5^. However, it forms shallow pits when reconstituted on lipid monolayers^5^ and does not bud the membrane of large unilamellar vesicles (LUVs)^6^. These results suggested that clathrin polymerization energy was not the dominant factor in controlling the shape of clathrin coats.

On the contrary, epsin^16^, a clathrin adaptor structurally similar to AP180, facilitates the clathrin assembly on membranes into highly curved coated buds^17^. Although less potent in clathrin polymerization, epsin has an ENTH domain containing an amphipathic helix-0 that wedges itself into the outer membrane leaflet, helping the build-up of curvature^17^. This supports the direct role of adaptors in curvature generation.

Lipid membranes are deformable, fluid surfaces^18^. To deform a membrane by scaffolding, the energetic gain upon coat polymerization must be at least equal to the energetic cost associated with membrane deformation. The clathrin polymerization energy has never been compared with membrane budding energy. This depends on membrane tension, rigidity and geometry of the bud. We thus questioned how parameters of membrane elasticity (that are, tension and rigidity) could affect clathrin polymerization.

## Results

### Membrane tension opposes clathrin polymerization

**Figure 1:**
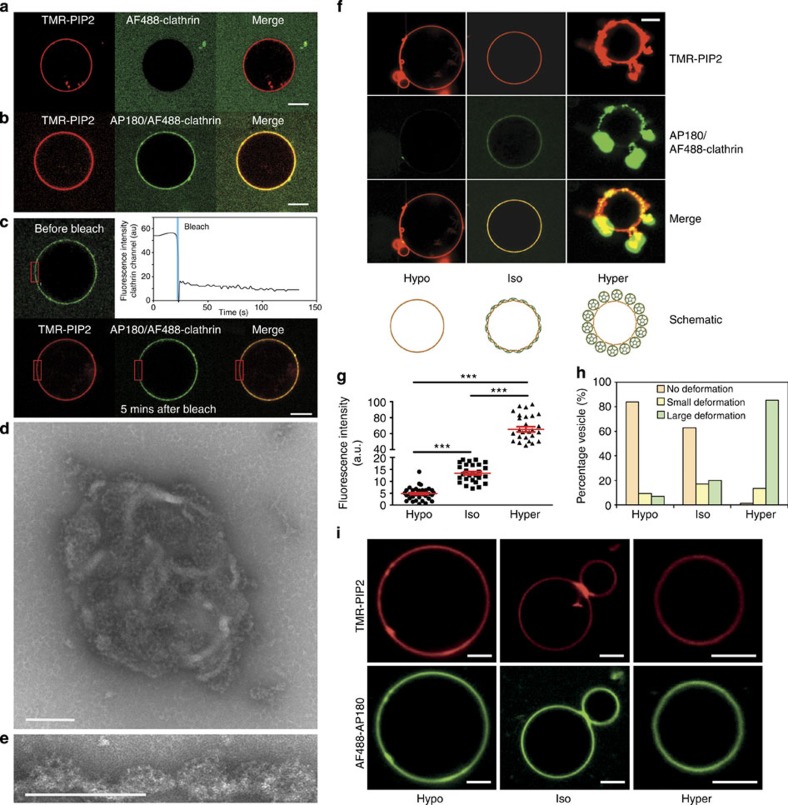
Reconstitution of clathrin polymerization on GUVs and effects of osmotic shocks. (**a**) GUVs labelled with 1 mol% TMR-PIP_2_ (red) incubated with AF488-clathrin alone (green) for 5 min. (**b**) Same conditions as in **a** but with AP180/AF488-clathrin. (**c**) Fluorescence recovery after photobleaching experiments of the AF488-clathrin coat on the membrane (see text). Scale bars, 5 μm (**a**–**c**). (**d**,**e**) TEM micrographs of GUVs coated with clathrin (scale bars, 200 nm (**d**) and 100 nm (**e**)). (**f**) GUVs labelled with TMR-PIP_2_ (red) were incubated with AP180/AF488-clathrin in three osmotic conditions: hypotonic (hypo), isotonic (iso) and hypertonic (hyper). Scale bar, 5 μm. (**g**) Mean fluorescence intensity of the clathrin binding (see Methods) for each osmotic condition, with s.e. (***P<0.0001 in *t*-test). (**h**) Statistics of vesicle appearance (large, small or no deformation) for the three osmotic conditions. (**i**) GUVs labelled with TMR-PIP_2_ (red) were incubated with AF488-AP180 under various osmotic conditions—hypotonic (hypo), isotonic (iso) and hypertonic (hyper). Scale bar, 5 μm. For all the fluorescence experiments, *n*=30–45 under each condition from at least three independent experiments.

**Figure 2:**
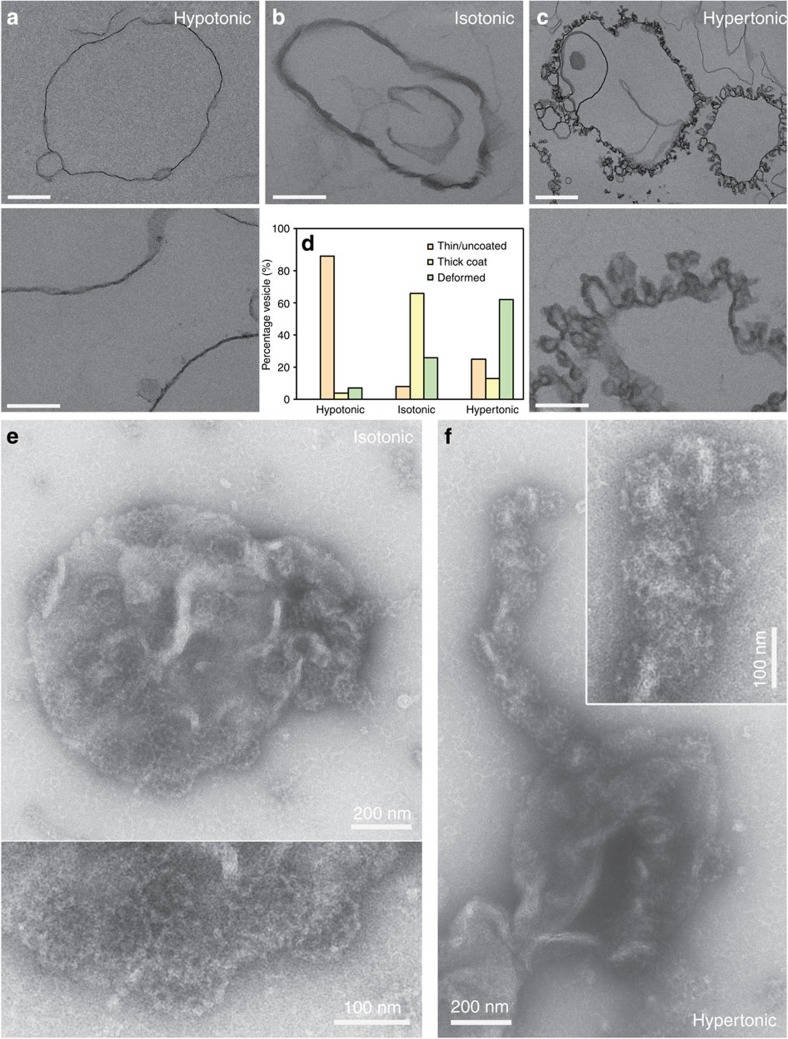
TEM images of GUVs incubated with AP180/clathrin under different osmotic conditions. (**a**) Hypotonic conditions. Scale bars, 1 μm (top row) and 200 nm (bottom row). (**b**) Isotonic conditions. Scale bar, 200 nm. (**c**) Hypertonic conditions. Scale bars, 1 μm (top row) and 200 nm (bottom row). (**d**) Statistics of GUVs appearance observed by TEM under different conditions. (**e**) Micrographs of clathrin lattices in isotonic conditions (scale bar, 200 nm) with a high-magnification image (scale bar, 100 nm (bottom)) and (**f**) micrographs of clathrin lattices in hypertonic conditions showing tabulation of the membrane (scale bar, 200 nm) with a high-magnification image (inset, scale bar, 100 nm). For electron microscopy experiments, *n*=372 (hypotonic), *n*=54 (isotonic) and *n*=134 (hypertonic) from at least three independent experiments.

To study the mechanism by which clathrin deforms the membrane, we reconstituted clathrin coats onto giant unilamellar vesicles (GUVs, 5–50 μm). We used GUVs containing 30% 1,2-dioleoyl-*sn*-glycero-3-phosphoserine (DOPS) and 10% PIP_2_ (see Methods), mimicking the composition of the inner plasma membrane leaflet. The use of 10% PIP_2_, although higher than physiological concentration, was used to rule out affinity differences between adaptors. Moreover, the physiological concentration of PIP_2_ within a clathrin-coated pit remains unknown. GUVs labelled with 1% of tetra-methyl rhodamine (TMR)-PIP_2_ were incubated with 0.5 μM purified AP180 and 0.4 μM purified clathrin containing 20 mol% of Alexa Fluor 488-labelled clathrin (AF488-Clathrin) (see Methods). As expected, clathrin bound to GUVs only in the presence of the AP180 adaptor (Fig. 1a,b) and brain PIP_2_ (Supplementary Fig. 1). After clathrin binding, clathrin appeared as a homogenous stain, colocalizing with the membrane signal (Fig. 1b). Clathrin formed a solid coat, as shown by the absence of recovery of coat fluorescence after photobleaching (Fig. 1c). Next, we studied the submicron structure of the clathrin coat using negative-stain electron microscopy (EM) of clathrin-coated LUVs. As previously reported^5^,^6^, we observed in most of the cases, small (a few tens of nm), clathrin assemblies (Figs 1d,e and 2e; Supplementary Fig. 2), covering the surface of the LUVs. The depth of these clathrin pits was difficult to address by negative stain, although many of them seemed rather shallow (Fig. 1e). Furthermore, the LUVs retained their round shape consistently with our confocal images of non-deformed GUVs. Importantly, all structures observed had limited depth (see Fig. 1c, and inset of Fig. 2e, isotonic; Supplementary Fig. 2). As clathrin/AP180 forms highly curved cages *in vitro* in the absence of membrane^15^, we hypothesized that the combined effect of tension and bending rigidity was responsible for the impairment of full budding by opposing the clathrin polymerization energy.

To confirm whether changes in membrane tension affected polymerization of clathrin, we transferred GUVs made in 208±1 mOsm sucrose solution to a clathrin/AP180 solution of varying osmolarity. We examined clathrin polymerization on GUVs under hypotonic (180±1 mOsm), isotonic (208±1 mOsm) and hypertonic (236±1 mOsm) external buffer conditions. In isotonic conditions, no optically visible deformations of GUVs were observed (Fig. 1f,h). We observed that under hypotonic conditions GUVs did not allow AP180/clathrin binding in most cases (Fig. 1f,g). We observed that fluorescent AP180 was binding efficiently under all osmotic conditions (Fig. 1i) with the same density (see Supplementary Fig. 3), even in the presence of clathrin. Furthermore, the binding of the terminal domain of clathrin (clathrin TD) to AP180 did not change significantly with the change of osmotic conditions (Supplementary Fig. 4). We thus concluded that increased membrane tension could totally impair clathrin polymerization. Also, as the surface density of the AP180 did not change in the presence of clathrin (see Supplementary Fig. 3), and since no deformation of the membrane was observed in presence of 0.5 μM AP180 alone in all osmotic conditions, we concluded that protein crowding had only a negligible effect, if any. While the 10% PIP_2_ concentration used provides more than enough binding sites to induce membrane deformation by crowding, the low affinity of AP180 ANTH domain for PIP_2_ (a few micromolars, see ref. 5), and the low AP180 concentration in our assay meant that not all binding sites were occupied. This accounts for the absence of membrane deformation mediated by AP180 crowding.

Strikingly, both clathrin and membrane fluorescence signals were strongly increased under hypertonic conditions (Fig. 1f,g). In many cases, long, highly coated membrane tubules emanated from the GUVs, suggesting that under hypertonic conditions, the membrane could be heavily deformed by the clathrin coat (Fig. 1f). We also observed five times more deformed GUVs in hypertonic conditions than in any other osmotic condition (Fig. 1h). These deformations appeared a few minutes after clathrin addition (see Supplementary Fig. 5a), which was consistent with the time it took to saturate the membrane with clathrin. AF488-clathrin/AP180-coated GUVs in isotonic conditions did not show more deformation when subjected to hypertonic shock (Supplementary Fig. 5b). These results support our hypothesis that GUV deformation was a result of clathrin polymerization.

To better characterize the shape of the membrane deformations, GUVs incubated with AP180/clathrin under varying osmotic conditions were fixed and processed for thin-section electron microscopy (TEM, see methods). Similar to the confocal images, TEM also revealed uncoated membranes when GUVs were incubated with proteins under hypotonic conditions (Fig. 2a,d). GUVs incubated under isotonic conditions revealed an electron-dense coat that did not cause observable deformations to the membrane (Fig. 2b,d). Furthermore, LUVs incubated with AP180/clathrin under isotonic conditions (see Fig. 2e) remained round shaped, as seen by negative-stain EM. On these images (see also Supplementary Fig. 2) the surface of the LUVs was covered with limited assemblies of clathrin, confirming that the electron-dense coat seen by TEM was indeed a proper clathrin coat. However, these images show that rather than a purely hexagonal flat lattice, the clathrin coat was constituted of densely packed shallow buds on the membrane (see Fig. 1d,e and Supplementary Fig. 2, monolayer and isotonic). Finally, GUVs incubated under hypertonic conditions showed an entirely budded surface, covered with an electron-dense coat (Fig. 2c,d). Coated membrane buds were observed with an average diameter of 65±5 nm, close to values of *in vitro* formed AP180/clathrin cages^15^. Furthermore, when LUVs were incubated in hypertonic conditions in the presence of AP180/clathrin, numerous LUVs showed a surface entirely coated with clathrin buds and long tubular extensions covered with many deep buds (see Fig. 2e; Supplementary Fig. 2, hypertonic). In summary, observations made by EM (both TEM and negative stain) were consistent with our confocal microscopy data (Fig. 1), and confirmed that AP180 and clathrin can deform the membrane in a membrane tension-dependent way.

### Membrane elastic energy against clathrin polymerization energy

**Figure 3:**
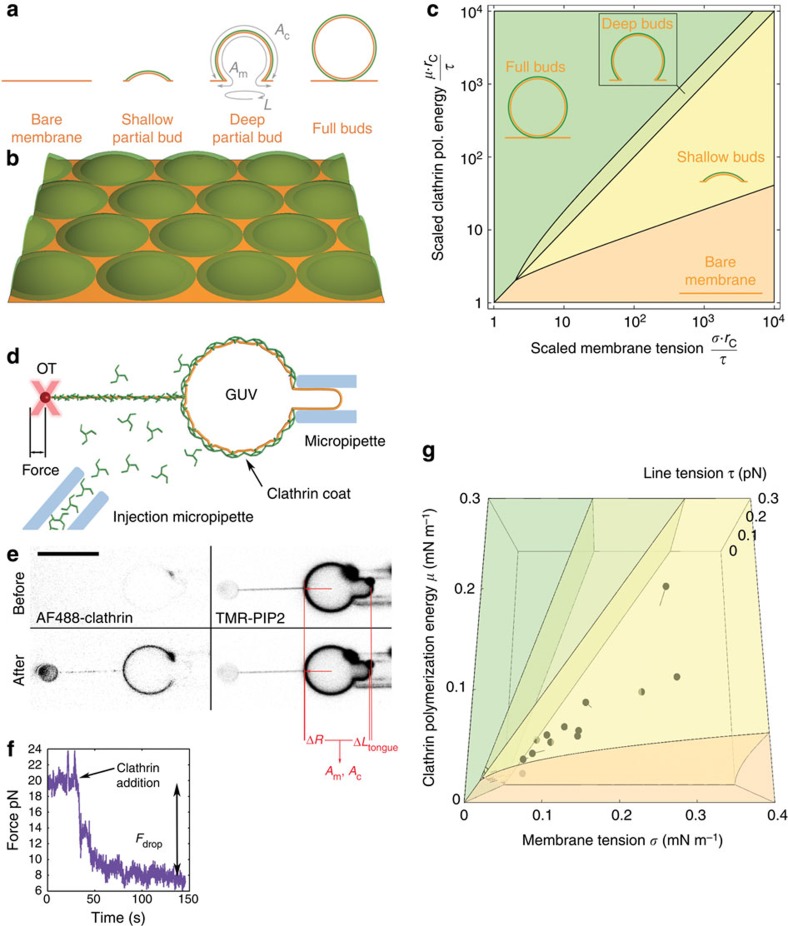
Model and clathrin polymerization energy measurements. (**a**) The four possible states of a single bud considered in the model: bare membrane (no clathrin binding), shallow partial budding, deep partial budding and full budding (yielding a closed bud). Parameterization of the bud is shown on the deep partial bud as discussed in the text. (**b**) Our mathematical model predicts the state of a membrane densely packed with contiguous buds. Our fitting of the model parameters (see main text) predicts a bud depth ≃2 nm. (**c**) Phase diagram predicted by our model, showing the predicted budding state as a function of the scaled tension and polymerization energy (logarithmic plot). (**d**) Experimental set-up for the measurement of the clathrin polymerization energy: OT, optical tweezers; GUV, giant unilamellar vesicle. (**e**) Confocal images of AF488-clathrin and TMR-PIP_2_ channel (right images) before and after clathrin injection (inverted contrast). Scale bar, 10 μm. (**f**) Plot showing tube force versus time during addition of clathrin. (**g**) Three-dimensional phase diagram with experimental data from **e** and **f** (linear plot). The clathrin polymerization energy was measured from 15 independent experiments.

To quantitatively test our hypothesis, we developed a theoretical model that describes the competition between clathrin polymerization, which favours full budding, membrane tension and rigidity, which favour planar membranes, to predict transitions between different possible bud shapes (see Fig. 3a). In the model, a flat membrane with tension *σ*, and bending rigidity *κ*, is in contact with a clathrin/AP180 solution at fixed concentrations. We denoted by *μ* the free energy gain associated with the polymerization of a unit area of clathrin coat. As membrane-bound clathrin coats are very rigid (bending rigidity *κ*_c_≃300 *k*_*B*_*T*^19^), we assumed that clathrin always polymerizes into spheres of fixed radius *r*_c_. In our model, tension cannot modify the coat’s radius, but it can limit clathrin polymerization to partial buds as illustrated in Fig. 3a,b. Accordingly, our model considers four possible bud states illustrated in Fig. 3a: (1) bare membrane: clathrin cannot polymerize, and the membrane remains uncoated; (2) shallow partial bud: clathrin polymerizes partially into buds that are less than a half sphere; (3) deep partial bud: clathrin polymerizes into a bud that is more than a half sphere but less than a full sphere and (4) full bud: a full spherical clathrin coat forms around the membrane bud. This coat is traversed by a small membrane tether of negligible energy^20^ that connects the membrane bud to the main membrane. This connection is the only difference between our full buds and free clathrin-coated vesicles.

This description is consistent with the partial coats observed by negative-stain EM (Fig. 2e; Supplementary Fig. 2). To support this geometrical picture, we measured the average size of partial buds grown on mica-supported bilayers by atomic force microscopy to find a height of 15–20 nm, and a diameter of 80–100 nm (see Supplementary Fig. 6). Subtracting the ~15 nm thickness of the clathrin coat, these measurements are consistent with membrane buds of ~30 nm radius, and a depth of a few nanometres as illustrated in Fig. 3b. This model is also consistent with our EM studies, where we observed bare membranes in hypotonic conditions (Fig. 2a), shallow buds in isotonic conditions (Fig. 2a,e) and deep buds in hypertonic conditions (Fig. 2a,f). These observations support our assumption that AP180/clathrin mostly polymerizes with a roughly constant radius of curvature *r*_c_. From our TEM pictures in hypertonic conditions (see above and Fig. 2), we thus concluded that *r*_c_=32.5 nm.

In the model, we decomposed the polymerization energy of clathrin into two contributions; 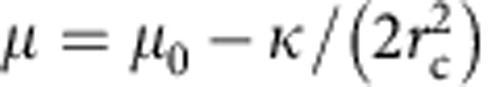, where *μ*_0_ is the (positive) clathrin-binding free energy and 
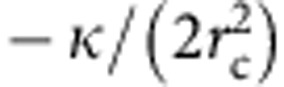
, the cost of bending the membrane into a bud. In addition, we introduced the coat’s line tension *τ*, describing the energetic cost of broken clathrin–clathrin bonds at the boundary of a partial bud. This leads to the following expression for the free energy of a single bud:


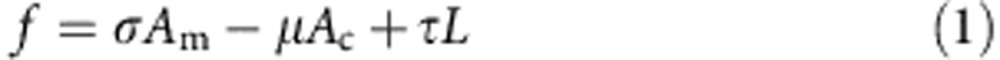


where *A*_m_ and *A*_c_ are the membrane and coat areas, respectively, associated with one bud, and *L* is the length of its rim (Fig. 3a). In this expression, *μ* is dimensionally equivalent to a force per unit length, and represents the net force that a clathrin coat applies on the membrane while polymerizing. The membrane tension *σ* and the bending rigidity *κ* oppose this force, while the coat’s line tension *τ* favours buds with short rims. To make predictions at the liposome level, we applied this theoretical framework to a densely packed assembly of coated buds (see Fig. 3b and Supplementary Discussion). Depending on the value of the parameters, we can analytically predict the shape of the clathrin buds (bare membrane, shallow/deep partial buds or full buds) as shown in the phase diagram Fig. 3c. Qualitatively, for low clathrin polymerization energies (*μ*≤2*τ*/*r*_c_), the membrane is either bare for high tensions (*σ*>*μ*) or fully budded if *σ*≤*μ*. These two regimes still exist at high polymerization energies (*μ*>2*τ*/*r*_c_), but with the added possibility of partial buds at moderate tensions. This high polymerization energy situation reproduces our above observations of GUVs under various osmotic conditions. We observed no polymerization for high tensions, and full budding for low tensions. Moreover, we do not observe purely hexagonal lattices (see Fig. 2, and also Supplementary Fig. 2), and thus interpret the continuous fluorescent staining of clathrin on GUVs (Fig. 1) in isotonic conditions as a dense coverage of contiguous shallow buds (see Figs 1e and 3b).

To validate our model, we measured membrane tension *σ* and clathrin polymerization energy *μ*. We used an *in vitro* micromanipulation assay (see Fig. 3d)^21^ to pull a membrane nanotube out of GUVs by means of a streptavidin bead held in optical tweezers. We modulated the membrane tension *σ* by aspirating the GUV in a micropipette. This technique allowed us to quantify and control tension in a way that cannot be achieved by osmotic shock. We measured the force *F* required to maintain the tube using optical tweezers. In the absence of clathrin, this force is given by^22^ 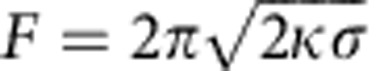, allowing us to measure^23^ the membrane bending modulus *κ*=13±5*k*_*B*_*T*. Once the tube was formed, a mix of AP180 and AF488-clathrin in isotonic buffer was injected using a second micropipette (see Methods and Fig. 3d). Clathrin binding was observed along the membrane tube and on the GUV surface. Clathrin GUV staining was homogeneous (Fig. 3e), consistent with the formation of a continuous flat coat as in Fig. 1a,b. Following clathrin polymerization, we observed a reduction of the total apparent GUV membrane area in 87% of all experiments, consistent with the sequestration of the membrane into clathrin-coated pits (see Fig. 3d,f; Supplementary Table 1). The 6% reduction of the apparent GUV area (see Supplementary Table 1) allowed us to calculate an average bud depth of 2 nm, which is consistent with our EM data. During clathrin polymerization on the GUV and the tube, we measured a drop in the tube force (see Fig. 3f). AP180 alone did not induce a significant force drop when binding to the tubes (see Supplementary Fig. 7). In cases where the apparent membrane area decreased, we used our mathematical model to infer the values of the parameters *μ* and *τ* from the decrease in apparent membrane area and tube force (Supplementary Discussion). The resulting values are given in Supplementary Table 1 and denoted by full circles in the phase diagram of Fig. 3g (colour code identical to Fig. 3c). In all cases, the measured values of *μ* and *σ* fell in the shallow bud regime, confirming our hypothesis that continuous staining of clathrin-coated GUVs in isotonic conditions were indeed contiguous shallow buds. The measured value of the clathrin polymerization energy per unit area was equal to 7.6±5.4 × 10^−5^ N.m^−1^, with *μ*_0_=1.0±0.5 × 10^−4^ N.m^−1^. We found *τ*=5.2±7.4 × 10^−2^ pN, well below values at which line tension is able to bud membrane domains on its own (typically 1 pN, see ref. 24). This supports our hypothesis that membrane bending is primarily due to clathrin polymerization and not due to the line tension.

**Figure 4:**
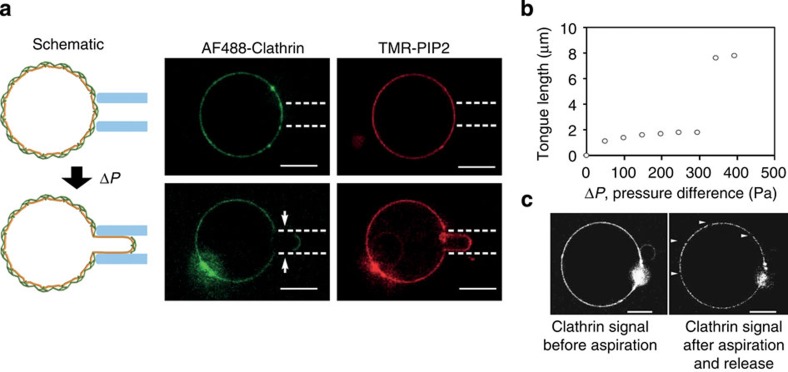
Clathrin coat rupture experiments. (**a**) Schematic and confocal images of AP180/AF488-clathrin-coated GUVs before and after being aspirated using a pipette. Arrows show the clathrin-uncoated part of the membrane tongue. (**b**) Length of the tongue versus aspiration pressure (Δ*P*). (**c**) AF488-clathrin before and after aspiration (tongue fully released). Arrowheads indicate cracks in the clathrin coat. Scale bars, 5 μm. The experiment was repeated 12 times to measure the average aspiration pressure Δ*P* for coat rupture.

The clathrin polymerization energy is the sum of two contributions: clathrin–clathrin interactions and clathrin–membrane interactions mediated by the adaptors, which we express as *μ*_0_=*μ*_c−c_+*μ*_c−m_. In the scaffolding mechanism, clathrin polymerization is the main driver of membrane deformation, implying that *μ*_c−c_ is much larger than *μ*_c−m_. To test this model, we estimated the relative importance of *μ*_c−c_ and *μ*_c−m_ by measuring the force required to rupture the clathrin coat. We aspirated clathrin-coated GUV using a micropipette of radius *R*_p_≃1 μm (Fig. 4a) in isotonic conditions. For moderate pressures, the vesicle retained its spherical shape as the coat prevented the deformation of the membrane. Upon increasing the aspiration pressure above Δ*P*^c^=231±26 Pa, we observed a sudden rupture of the coat along the rim of the pipette followed by marked membrane tongue elongation (Fig. 4a,b). The resulting cracks within the coat were visible both during a prolonged aspirated state and after releasing the aspiration (Fig. 4c). We reasoned that rupturing the coat in this way disrupted the clathrin–clathrin bonds while leaving the clathrin–membrane interactions unaffected, thus allowing us to estimate *μ*_c−c_ independently of *μ*_c−m_. From Δ*P*^c^ and *R*_p_, we estimated *μ*_c−c_=1.1±0.1 × 10^−4^ N.m^−1^ (see Supplementary Note 1). This value is indistinguishable from *μ*_0_=1.0±0.5 × 10^−4^ N.m^−1^, confirming that clathrin–clathrin interactions are the predominant interactions responsible for clathrin coat’s assembly as in the scaffolding model. Multiplying *μ*_c−c_ by the specific area *a*≃800 nm^2^ occupied by a triskelion (see Supplementary Note 2) yields the binding energy per triskelion *aμ*_c−c_=23±3*k*_*B*_*T*. Our value of the binding energy is consistent with a previous theoretical estimate based on the critical concentration of clathrin polymerization^25^, *aμ*_c−c_=42 *k*_*B*_*T*, and with estimates coming from computational assembly statistics^26^, where *aμ*_c−c_=23 *k*_*B*_*T*. Also, it is too large to allow for internal rearrangements of the clathrin lattice once polymerized, implying that clathrin buds directly polymerize into curved structures rather than going through a hexagonal flat coat intermediate.

### Espin facilitates clathrin polymerization

**Figure 5:**
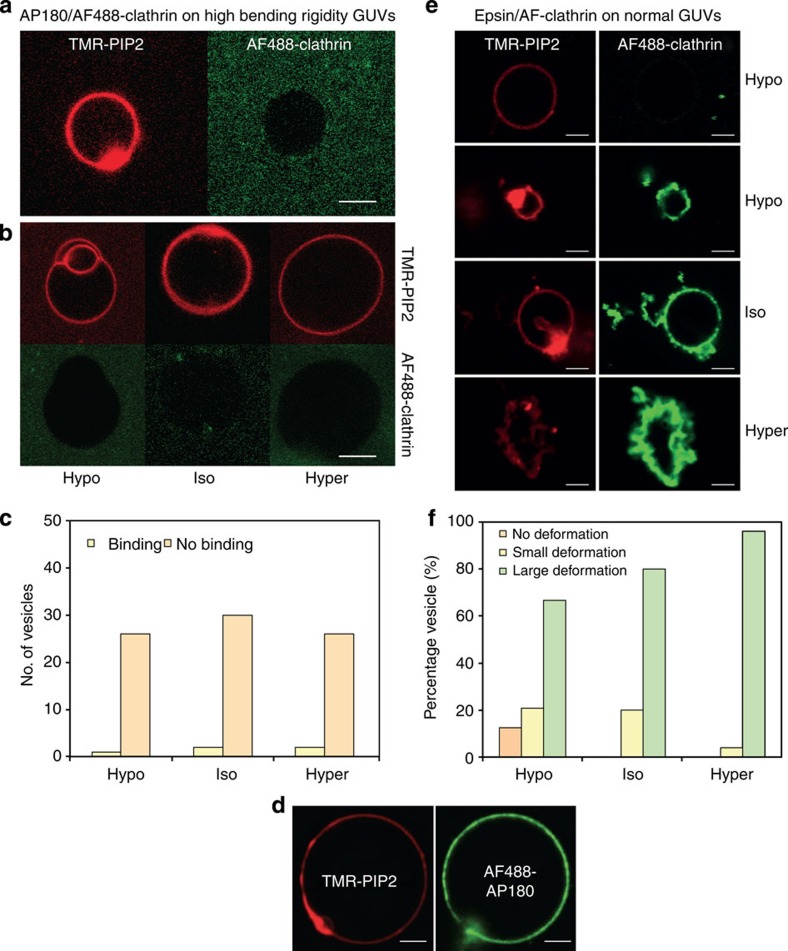
Effect of high bending rigidity and epsin on the clathrin budding abilities. (**a**) AF488-clathrin/AP180 does not bind to GUVs with high bending rigidity (see Methods) under isotonic conditions. (**b**) AF488-clathrin does not bind to high bending rigidity GUVs in hypo-, iso- and hypertonic conditions. (**c**) Number of vesicles showing AP180/clathrin binding or no binding under various external buffer conditions. (**d**) A high binding rigidity did not preclude binding of AP180. (**e**) AF488-clathrin binding and membrane deformation in the presence of unlabelled epsin were observed under varying osmotic conditions, except in the hypotonic conditions where a fraction of the vesicles remained uncoated. (**f**) Percentage of vesicles with no, small or large deformation after AF488-clathrin/epsin binding. Scale bar, 5 μm. For all the fluorescence experiments, *n*=30–45 under each condition from at least three independent experiments.

To further validate our model, we used these results to predict the shape of clathrin coats by changing parameters. We tested this prediction experimentally by investigating the predicted role of the bending rigidity in inhibiting budding. The expression 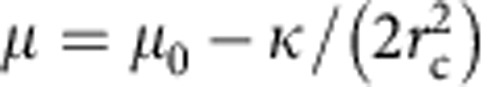 implies that an increased *κ* should reduce the value of the polymerization energy *μ*. We used GUVs of higher bending rigidity obtained by adding sphingolipids and cholesterol (60% brain sphingomyelin (BSM), 30% DOPS, 10% PIP_2_, with 50% cholesterol) to increase the bending rigidity to *κ*=51±20*k*_*B*_*T*. This decreased the average value of *μ* by more than an order of magnitude (*μ*_stiff_=3.7 × 10^−6^ N.m^−1^), implying a marked decrease of the maximum tension allowing clathrin assembly to 3.5 × 10^−6^ N.m^−1^. Consistent with our expectation, clathrin failed to bind to a vast majority of liposomes (Fig. 5a) under isotonic conditions despite significant binding of AP180 (Fig. 5d). We further tested whether clathrin polymerization could be restored by changing membrane tension, but did not observe any clathrin binding neither under hypotonic nor hypertonic conditions (see Fig. 5b,c), further showing the influence of membrane bending rigidity on clathrin polymerization.

To further test our model, we predicted that any adaptors with membrane-deforming properties should assist budding. We thus studied the polymerization of clathrin on GUVs as mediated by epsin. We found that epsin binds to liposomes under all tested membrane tensions (Supplementary Fig. 8) without causing observable deformations, as does AP180. When GUVs were incubated with the unlabelled epsin and AF488-clathrin under hypotonic conditions, 70% of the vesicles showed membrane deformations (Fig. 5e,f). Significant membrane deformation and tubulation were also observed under isotonic conditions. Likewise, under hypertonic conditions the GUVs were highly deformed (Fig. 5d). Thus the membrane-deforming properties of epsin facilitated clathrin polymerization to such an extent that it was able to bind to GUVs even under unfavourable membrane tension conditions.

## Discussion

In this study, we showed that the shape of clathrin coats is controlled by membrane tension. This mechanism is based on counteracting clathrin polymerization by membrane tension, which hinders the closure of the clathrin-coated pits: at low membrane tension, deep buds can form, whereas intermediate or high tensions will only result in partial budding. We measured the clathrin polymerization energy and found values that are in the range of *in vivo* membrane tension values (10^−5^–10^−4^ N.m^−1^—see refs 27, 28, 29). Thus, *in vivo* values of membrane tension cover the shape transition from a shallow to a deep budded coat, supporting a physiological control of clathrin budding through membrane tension. It is consistent with the fact that clathrin-mediated endocytosis is delayed in cells that have higher membrane tension and require actin for completion^30^,^31^.

We imagine that at the structural level, membrane tension does not allow for the correct binding angle between arms of the triskelia, impairing their correct association.

We also found that increased membrane rigidity opposes clathrin budding. It is consistent with the fact that clathrin-mediated endocytosis is slower at the apical pole of epithelia, where the membrane is more rigid than at the basal pole^32^. We also found that the membrane-deforming ability of epsin favours clathrin-mediated budding, even when the membrane’s elastic parameters (namely tension and rigidity) are not favourable. This indicates that the different actors that can generate membrane curvature (wedge insertion, crowding or local forces exerted by the cytoskeleton) can act synergistically to deform the membrane: the role of adaptors and actin in clathrin-induced membrane deformation has been studied extensively. However, conclusions about whether they are required or sufficient for membrane deformation proved themselves difficult to generalize to all biological systems. Our results support a modulatory role of each adaptor and actin depending on the difficulty of deforming the membrane^31^: in an extreme case where the membrane is very stiff and tension is high, all partners are required, whereas they become dispensable in intermediate conditions.

For energetic^25^, kinetic and biochemical reasons^33^, it seemed more favourable to consider that clathrin polymerizes directly into its final curved shape. However, explaining the various shape of clathrin coats has for long been a matter of debate: it was proposed^12^ and analysed theoretically^26^,^34^ how the clathrin lattice could be reorganized to change shape once polymerized. Another possibility is that the clathrin coat is soft enough once polymerized to deform elastically under increasing tension. However, measurement of the clathrin-coated membrane bending rigidity showed it is about 10 times more than free lipid membranes, which accounts for less than 5% change in size by elastic deformation (theory not shown). Moreover, our model and findings favour the direct polymerization of clathrin in a curved lattice with AP180. They also account for how the shape of the bud could be modulated by membrane tension. Our findings are compatible with the fact that partial knockdown of clathrin generates shallow pits *in vivo*^35^, as our model predicts a decrease in clathrin’s cytosolic concentration would result in a lowering of its polymerization energy *μ*. Similar shallow coats were also observed with both H_6_-ΔANTH-AP180 and full-length H_6_-AP180, together with clathrin in a recent reconstitution study suggesting that clathrin polymerization alone could drive membrane bending^13^. The polymerization of other membrane coats has also been shown to depend on tension. Such examples include the shape of Coat Proteins I (COPI) coats that change from flat to a budded state when tension is decreased^36^, and caveolae that can be disrupted by increased membrane tension^28^. More generally, actin plays a critical role in the clathrin bud formation, in all cell types that have been studied, from yeast^37^ to mammals^28^,^29^. Actin is the main membrane tension regulator in eukaryotic cells. Its role in clathrin-mediated endocytosis thus may indicate that membrane tension is a general regulator of clathrin-mediated endocytosis.

## Methods

### Materials

1,2-dioleoyl-*sn*-glycero-3-phosphocholine (DOPC), 1,2-dioleoyl-*sn*-glycero-3-phosphoethanolamine (DOPE), L-α-phosphatidylinositol (liver PI), DOPS, PIP_2_, BSM, di-stearoyl phosphatidyl ethanolamine-PEG(2000)-biotin and cholesterol were purchased from Avanti Polar Lipids. Fluorescent lipid bodipy-tetra-methyl-rhodamine-PIP_2_ (TMR-PIP_2_) were purchased from Echelon Biosciences. Two lipid compositions were used—(1) 25% DOPC+30% DOPE+5% liver PI+30% DOPS+10% PIP_2_ supplemented with 15% cholesterol and 1% TMR-PIP_2_; (2) 60% BSM+30% DOPS+10% PIP_2_+1% TMR-PIP_2_ supplemented with 50% cholesterol. streptavidin-coated polystyrene beads (3.05 μm) used for nanotube pulling experiments were purchased from Spherotech. Clathrin was extracted from fresh porcine brains and purified as described in ref. 15. Clathrin was labelled with Alexa Fluor 488 C5 maleimide (Invitrogen). AP180 was expressed in BL21/DH3α cells and purified by glutathione S transferase pull-down and glutathione S transferase cleaved by PreScission protease. AP180 was labelled with Alexa Fluor 488 C5 maleimide (Invitrogen). Epsin was purified from cells infected with baculovirus as described in ref. 5 and labelled with Alexa Fluor 488 C5 maleimide (Invitrogen). All proteins were aliquoted in GTPase buffer (HEPES 20 mM pH=7.4, NaCl 100 mM and MgCl_2_ 1 mM) and stored at −80 °C.

### Preparation of GUVs

The GUVs were composed of 25% DOPC, 30% DOPE, 5% liver PI, 30% DOPS, 10% PIP_2_, 15% cholesterol and 1% TMR-PIP_2_ (fluorescent lipid) in chloroform/methanol at a concentration of 2 mg ml^−1^. The lipid mix was dried under N_2_ flux and further dried under vacuum for 1 h before being resuspended in pure chloroform. To obtain a good yield of GUVs containing highly negatively charged lipids, the electroformation of GUVs was performed as described in ref. 21. Briefly, a uniform smear of 10 μl of lipid mix at 2 mg ml^−1^ was first dried on conductive indium-tin oxide-coated glass (Präzisions Glas and Optik GmbH) for 30 min at 60 °C, followed by drying under vacuum for at least 1 h. The lipid film was then rehydrated in a sucrose solution at 208±1 mOsm and GUVs were allowed to grow for 1 h under a sine voltage (900 mV, 10 Hz). For iso-osmotic experimental conditions, a sucrose solution of equal osmolarity as the protein solution was used, while for hypertonic and hypotonic conditions, more concentrated or less concentrated (respectively) sucrose solutions were used.

### Confocal microscopy

An open homemade observation chamber was incubated with a 2 mg ml^−1^ casein solution for 15 min to avoid adhesion of GUVs to the coverslip. The chamber was then rinsed twice with GTPase buffer (see above) before injecting 180 μl of the same buffer containing the proteins. The final concentrations of AP180 and clathrin were 0.5 and 0.4 μM, respectively. The protein mix was allowed to equilibrate in the chamber, followed by injection of 4–5 μl of GUVs. Imaging was carried out on a Nikon confocal microscope. Identical laser power and gain settings were used during the course of all experiments.

### Electron microscopy

An excess of GUVs in different osmotic environments was incubated with protein mix and visualized by confocal fluorescence microscopy for binding of AP180/clathrin. The protein-bound GUVs were fixed with 4% glutaraldehyde, embedded in 2% agar to increase the sedimentation of the liposomes and spun for 5,000 r.p.m. for 3–4 min to pellet down the sample. The sample was treated with 0.4 M Millonig’s buffer (sodium phosphate (monobasic)+NaOH) containing 2% osmium tetroxide for 1 h and rinsed with ddH_2_O. The sample was stained with 0.25% uranyl acetate overnight and rinsed with ddH_2_O followed by sequential dehydration in 30, 50, 70 and 90% ethanol for 5–10 min. The last dehydration step was carried out three times in absolute ethanol for 30 min each. The sample was then washed with propylene oxide twice for 10 min followed by incubation in epon-propylene oxide (1:1) for 1 h. Finally, the sample was treated with 100% epon overnight at room temperature before being embedded in 1 ml of epon resin mix followed by curing at 65 °C for at least 24 h. Ultrathin sectioning was performed using a microtome (Leica Ultracut) at a cutting angle of 6°. Sections were put on glow-discharged carbon-coated formvar grids and viewed with a Tecnai G2 Sphera (FEI) electron microscope at an acceleration voltage of 160 kV and an exposure time of 1,000 ms. Also, for negative-stain EM observations, LUVs were first incubated with AP180/clathrin in suspension for 10–15 min and then adsorbed onto Formvar-coated EM grids. The samples were fixed and negatively stained with 2% uranyl acetate before visualization.

### Clathrin polymerization energy measurements

GUVs were aspirated in a micropipette controlled by a motorized micromanipulator (Sutter MP225) and a custom-made hydraulic system to control the aspiration pressure Δ*P* and to set the membrane tension: 
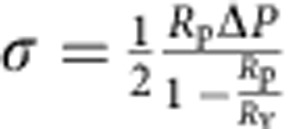
 where *R*_p_ and *R*_v_ are the radii of the pipette and GUV, respectively. A membrane nanotube was pulled out of a GUV whose membrane was attached to a streptavidin-coated bead (3.05 μm diameter, Spherotec) held in a fixed optical trap. The optical trap was custom-made with an ytterbium fibre laser focused through a 100 × 1.3 numerical apperture oil immersion objective. The force *F* exerted on the bead was calculated from the Hooke’s law: *F*=k*Δ*x*, where *k* is the stiffness of the trap (*k*=360 pN μm^−1^ W^−1^) calibrated by the viscous drag method^38^ and Δ*x* the displacement of the bead from its equilibrium position in the optical trap measured with a custom-made video tracking software. AP180 and clathrin were injected close to the nanotube using a second micropipette with a typical radius of 10 μm controlled with a hydraulic micromanipulator (Narishige). Nanotubes were visualized simultaneously by brightfield imaging (Pixelink camera) and by dual-colour confocal microscopy (*λ*_1_=488 nm and *λ*_2_=543 nm) on an inverted Nikon eclipse Ti microscope. The clathrin polymerization energy was measured for increasing values of set membrane tensions.

### Image analysis

Images were analysed and processed with ImageJ. The fluorescence signal, *F*_vesicle_ along the equatorial plane of GUVs was quantified by measuring the average fluorescence of the membrane using the OvalProfile plugin of ImageJ. The background fluorescence of the unbound protein, *F*_background_ was measured in an area close to the vesicle. Fluorescence intensity of the vesicle was denoted as *I=*(*F*_vesicle_*−F*_background_). Same illumination and detection settings were used for all the experimental conditions. Electron micrographs were manually analysed using ImageJ to measure the average bud diameter of 65±5 nm under hypertonic conditions.

### Atomic force microscopy on supported bilayer

Ten μl of a 10-mg ml^−1^ lipid mixture was dropped onto a circular glass coverslip, air dried and kept under vacuum for at least 1 h. The dried lipid smear was hydrated with 90 μl of GTPase buffer for 30 min, followed by rinsing to peel off the membrane regions that did not adhere to the surface. Two μl of the protein mix (AP180+clathrin) was incubated with the adhered membrane sheet for 30 min, followed by rinsing with GTPase buffer to wash the unbound protein. The protein-binding membrane sheet was then fixed with 4% glutaraldehyde and 1.8% osmium tetroxide for 1 h, followed by washes with GTPase buffer. The sample was then dehydrated by series of ethanol washes (30%, 50%, 70% and 90% ethanol for 5 min each) and further kept overnight under absolute ethanol. The dried sample was then imaged using the Multimode V AFM (Veeco) in tapping mode. Pointprobe Plus tips (Nanosensors, Neuchatel, Switzerland) with non-contact high frequency (*C*=42 N.m^−1^, *f*_o_=330 kHz) were used for scanning the image.

## Author contributions

A.R. and M.S. designed the research. M.S., S.M., A.H. and A.R. carried out research. J.M. provided technical assistance in protein purification. M.L. developed the theoretical model. M.S., M.L. and A.R. analysed the data. M.S., M.L. and A.R. wrote the paper.

## Additional information

**How to cite this article:** Saleem, M. *et al*. A balance between membrane elasticity and polymerization energy sets the shape of spherical clathrin coats. *Nat. Commun.* 6:6249 doi: 10.1038/ncomms7249 (2015).

## Supplementary Material

Supplementary Information Supplementary Figures 1-8, Supplementary Table 1, Supplementary Notes 1-2 and Supplementary Discussion
