# Supplementary material for: A balance between membrane elasticity and polymerization energy sets the shape of spherical clathrin coats
Source: Nat Commun. 2015 Feb 19;6:6249. doi: 10.1038/ncomms7249 (PMC4346611; doi:10.1038/ncomms7249)
Supplement: Supplementary Information — Supplementary Figures 1-8, Supplementary Table 1, Supplementary Notes 1-2 and Supplementary Discussion [file ncomms7249-s1.pdf]

## Supplementary Information

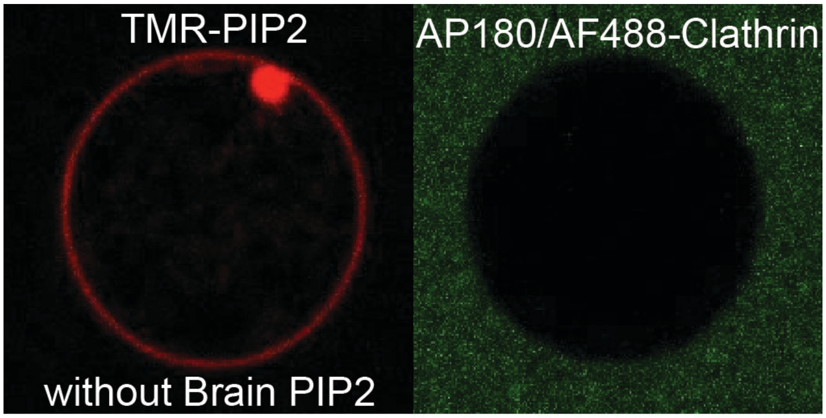


**Supplementary Figure 1:** AP180/AF488-Clathrin does not bind to GUVs containing 1% TMR-$PIP_2$ but no Brain $PIP_2$.

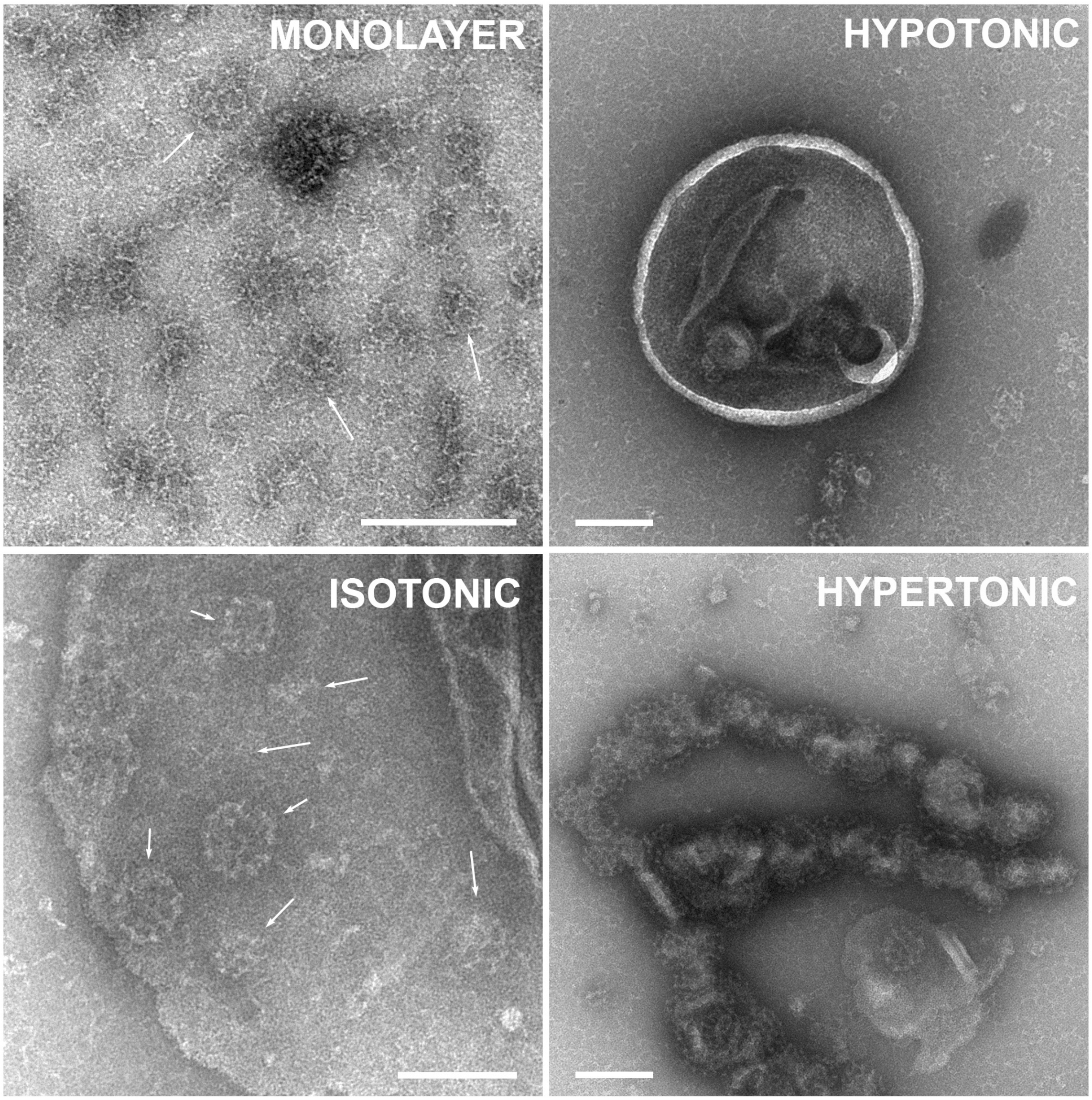
MONOLAYER
HYPOTONIC
ISOTONIC
HYPERTONIC

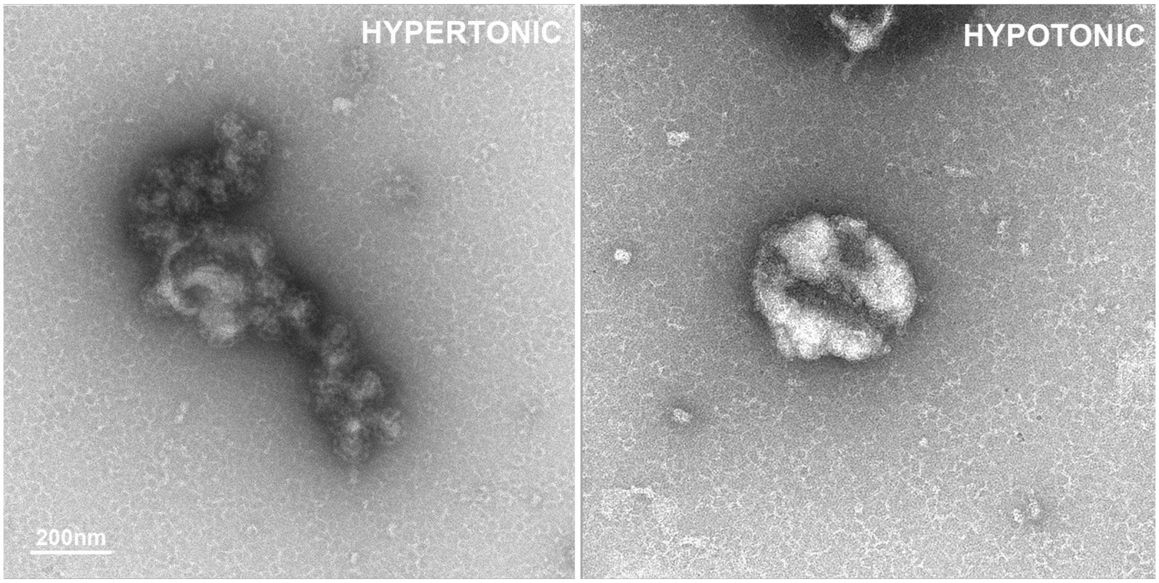


**Supplementary Figure 2: AP180/Clathrin assemblies as seen by negative stain.** On a Langmuir lipid monolayer AP180/clathrin were found to form small assemblies (white arrows) that are not forming a purely flat, hexagonal lattice. The same structures (white arrows) are seen in isotonic conditions on LUVs, whereas no clathrin structures are seen on LUVs in hypotonic conditions. In hypertonic conditions, numerous clathrin coated membrane tubules are seen, decorated with many deep buds. Bars, 200nm.

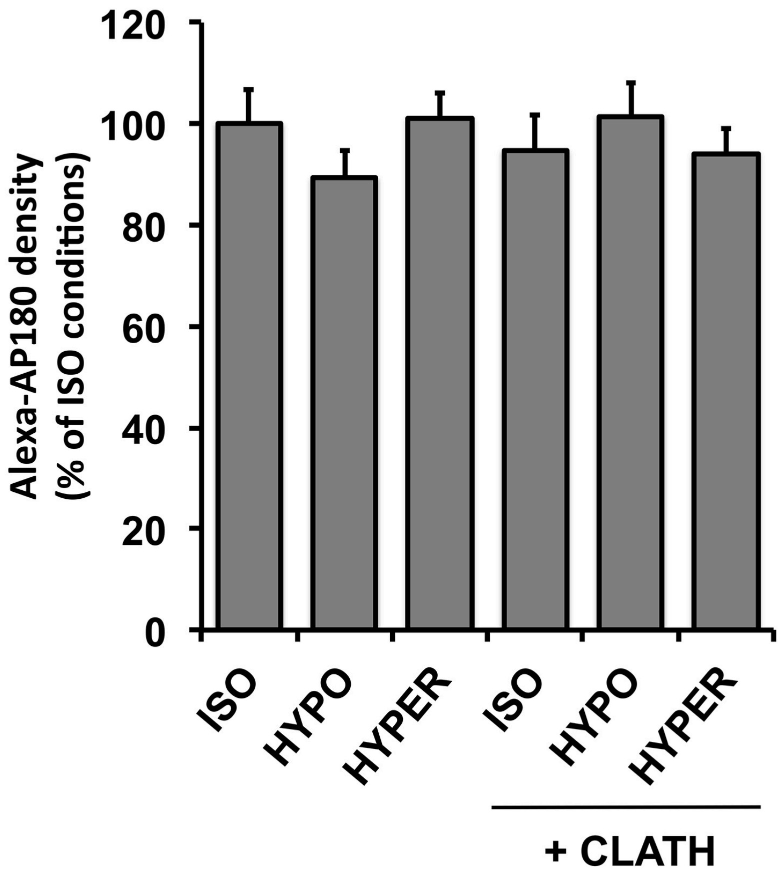


**Supplementary Figure 3:** Alexa-AP180 density on GUVs as measured by the ratio of fluorescence intensities of Alexa488 (AP180) and Tetra Methyl-Rhodamine signals ($PIP_2$) normalized to the isotonic conditions. ISO: Isotonic, HYPO: Hypotonic, HYPER: Hypertonic. + CLATH, same conditions +0.4 micromolar clathrin.

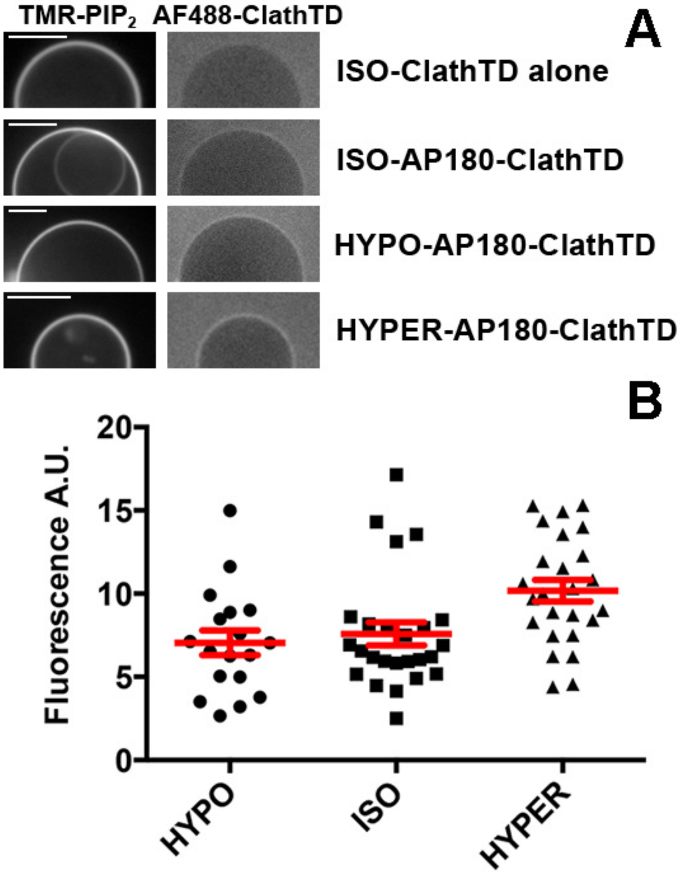


**Supplementary Figure 4:** Binding of Clathrin Terminal Domain to membrane in the presence of AP180. (A) Binding of AlexaFluor488 labeled clathrin Terminal Domain (AF488-ClathTD) to GUVs labeled with TMR-PIP2. When AP180 is absent, no detectable signal is seen on the vesicles. In other conditions (ISO for isotonic, HYPO for Hypotonic and HYPER for Hypertonic, see text) weak but specific binding of AF488-ClathTD is visible. (B) Fluorescence quantification of the binding of AF488-ClathTD to AP180 coated vesicles in the three osmotic conditions.

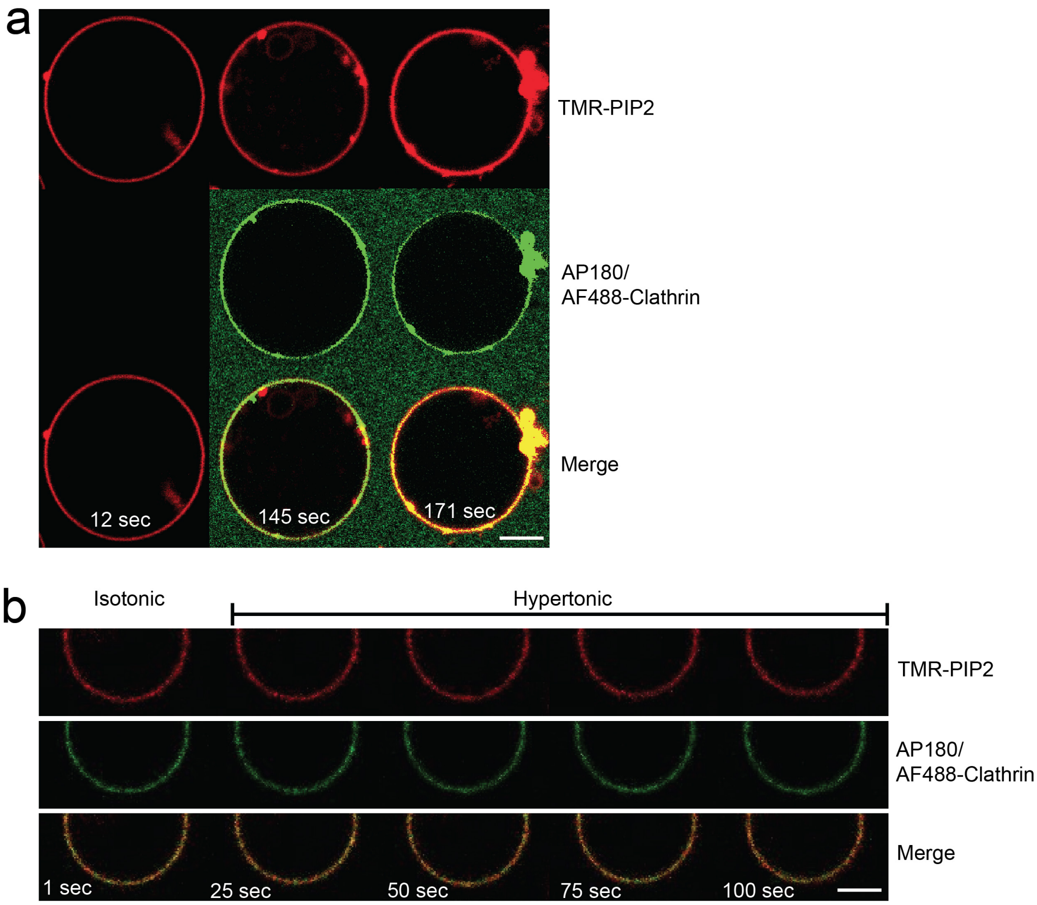


**Supplementary Figure 5: a)** GUVs under hypertonic conditions incubated with AP180/AF488-Clathrin. Confocal micrographs of the equatorial plane (lipid, protein and merged channels) suggest that significant perturbations on the outer surface of the membrane occur only after the binding of protein (145 seconds onwards). **b)** GUVs bound by AP180/AF488-Clathrin under isotonic conditions exposed to hypertonic conditions. The confocal snapshots of the equatorial plane (lipid, protein and merged channels) show no visible deformations occur due to the change of osmotic condition subsequent to clathrin polymerization. Scale bar, 5 μm.

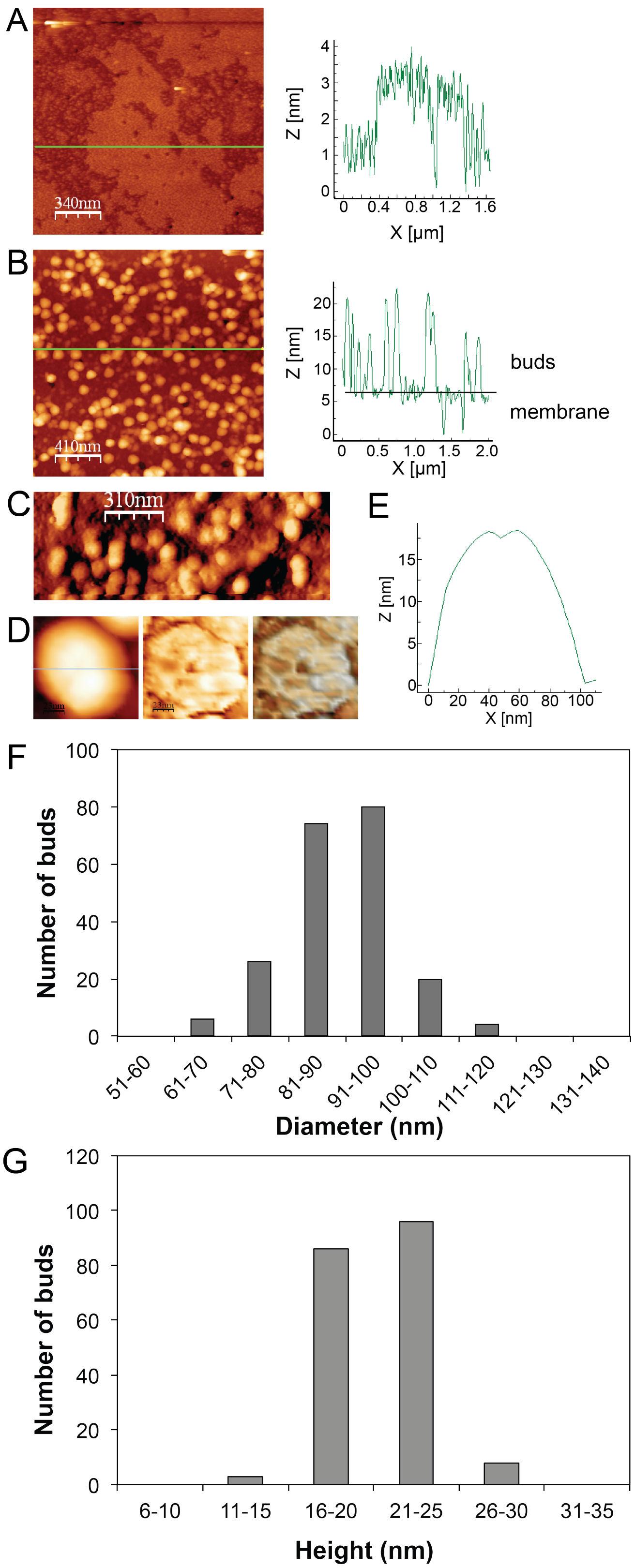


**Supplementary Figure 6: Atomic force microscopy of Clathrin structure on mica-supported bilayers.** (A) Topography of a supported membrane sheet showing a height profile of 4 nm. (B) Topography of a supported membrane incubated with AP180/clathrin showing several protruding structures as shown in image and height profile (right hand side). (C) High resolution atomic force microscopy images showing the hemispherical buds with a partially resolved clathrin lattice with a height of ~20 nm and diameter of ~90 nm. (D) Height, phase and 3D textured images showing a single bud. (E) Height profile of the single bud shown in D. (F, G) Diameter and height distributions for the observed protrusions.

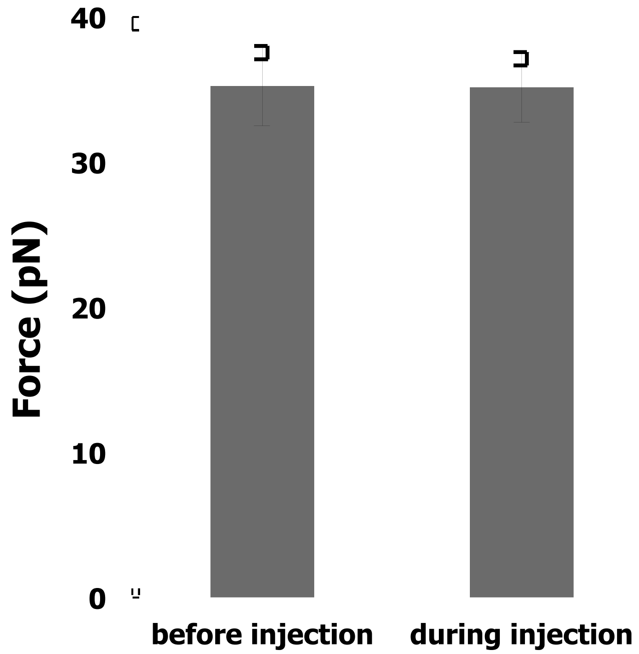


**Supplementary Figure 7. Force generated by AP180 binding onto the membrane nanotube.** Force on the membrane nanotube before and after the injection of AP180 does not vary significantly.

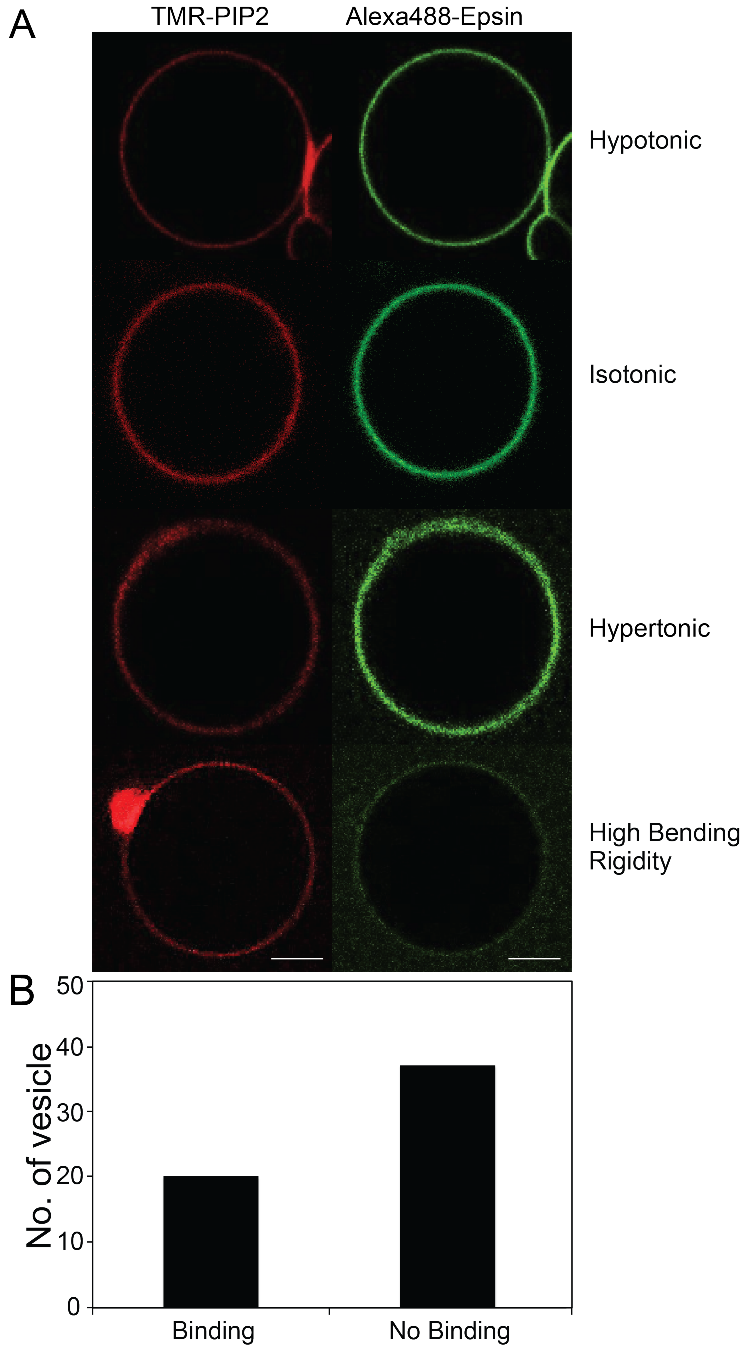


**Supplementary Figure 8. Epsin binding to membrane under several osmotic conditions.** (A) Alexa 488-Epsin binds as AP180 to giant liposomes under all varying ranges of membrane tension. Epsin also binds to membranes with high bending rigidity although weakly. (n > 45 vesicles for each case) (B) Epsin binding observed for high bending rigidity liposomes. (Scale bar, 5 μm)

**Supplementary Table 1:** Fitted Parameters

| vesicle number | σ ($Nm^{-1}$) | ΔA ($\mu m^2$) | fitted μ ($Nm^{-1}$) | μ0 ($Nm^{-1}$) | fitted τ (N) |
|---|---|---|---|---|---|
| 1 | 4.53E-05 | -9.7E-02 | | | |
| 2 | 3.68E-04 | -4.6E-02 | | | |
| 3 | 6.81E-05 | 1.77E-01 | 3.94E-05 | 6.31E-05 | 1.68E-13 |
| 4 | 5.32E-05 | 2.51E-02 | 5.19E-05 | 6.16E-05 | 3.43E-16 |
| 5 | 1.29E-04 | 3.57E-02 | 6.30E-05 | 1.02E-04 | 3.01E-14 |
| 6 | 1.49E-04 | 1.29E-01 | 9.11E-05 | 1.10E-04 | 1.98E-13 |
| 7 | 1.26E-04 | 1.49E-02 | 5.65E-05 | 8.58E-05 | 8.27E-15 |
| 8 | 1.03E-04 | 3.63E-02 | 6.74E-05 | 8.57E-05 | 1.66E-14 |
| 9 | 3.98E-05 | 1.97E-01 | 3.08E-05 | 5.92E-05 | 6.35E-14 |
| 10 | 3.14E-05 | 1.75E-02 | 1.29E-05 | 5.52E-05 | 2.84E-15 |
| 11 | 7.92E-05 | 3.18E-02 | 4.93E-05 | 8.54E-05 | 1.14E-14 |
| 12 | 7.14E-05 | 2.76E-02 | 5.78E-05 | 7.94E-05 | 4.18E-15 |
| 13 | 2.40E-04 | 6.95E-03 | 1.10E-04 | 1.39E-04 | 4.91E-15 |
| 14 | 3.05E-04 | 4.56E-03 | 1.29E-04 | 1.64E-04 | 3.53E-15 |
| 15 | 2.77E-04 | 1.22E-01 | 2.23E-04 | 2.38E-04 | 1.70E-13 |
| **average** | **1.29E-04** | **6.35E-02** | **7.56E-05** | **1.02E-04** | **5.25E-14** |
| **standard deviation** | **9.08E-05** | **6.76E-02** | **5.44E-05** | **5.19E-05** | **7.43E-14** |

**Supplementary Note 1:**
**Calculation of the rupture energy of clathrin:**
To relate $\Delta P^c$ to the clathrin-clathrin bond strength, we denote the force applied on the coat by the aspiration as $\pi R_p^2 \Delta P$ (pressure times area of the patch in the pipette). For a non-broken coat, this force is couteracted by the contact forces at the rim of the pipette, which are evenly distributed along the contact line of length $2\pi R_p$. Thus, each unit length of coat is subjected to a shearing force equal to $\pi R_p^2 \Delta P/(2\pi R_p) = R_p \Delta P/2$. At coat rupture, this expression is equal to the force per unit length, $\mu_{c\text{-}c}$, characteristic for the clathrin-clathrin bonds. Numerically, we use $R_p = 1\mu m$, implying $\mu_{c\text{-}c} = 1.1 \pm 0.1 \times 10^{-4}\, Nm^{-1}$.

**Supplementary Note 2:**
**Calculation of the clathrin-clathrin binding energy:**
The side of a clathrin hexagon is $s = 25\text{ nm}$, hence its area is $A = (3\sqrt{3}\, s^2)/2 = 1600\text{ nm}^2$. There are 6 triskelia per hexagon, but each is shared between 3 hexagons, thus there are 2 triskelia per hexagon on average. Hence the specific area of a triskelion is $a = A/2 = 800\text{ nm}^2$. As $\mu_{c\text{-}c} = 1.1 \pm 0.1 \times 10^{-4}\, Jm^{-2}$, the lateral interaction energy per triskelion is $a \times \mu_{c\text{-}c} = 8 \times 1.1 \times 10^{-20}\text{ J} = 23 \pm 3\, k_B T$.

Supplementary Discussion

# Supplemental mathematical modeling

Here we describe our model for the determination of the shape of clathrin-coated buds as a function of the membrane's mechanical parameters in the presence of a large (non-limiting) amount of the adaptor AP180. We first consider an infinite, planar membrane with tension $\sigma$. The membrane is put in contact with a clathrin solution of fixed concentration, which favors the assembly of clathrin buds of fixed radius $r_c$ on the membrane. Consistent with the free energy per bud introduced in Eq. (1) of the main text, we write the free energy per unit surface area of the initially flat membrane as

$$f = \frac{\sigma A_m - \mu A_c + \tau \ell}{A_p}, \tag{S1}$$

where $\mu = \mu_0 - \kappa/(2r_c^2)$ is the free energy gain per unit area upon clathrin polymerization – here $\mu_0$ is related to the concentration of the clathrin solution. In Eq. (S1), $A_m$ and $A_c$ respectively denote the membrane and coat areas associated with a single bud, $\ell$ is the length of the coat-bare membrane interface and $A_p$ is the projected area of the bud [Fig. 1(a)].

We assume that our system is at equilibrium, implying that the system selects the bud shape that minimizes the free energy of Eq. (S1). In Sec. , we describe the various acceptable morphologies for the system and the associated free energies. In Sec. , we compare these energies and determine the one that dominates the system depending on its parameters. Finally, in Sec. , we describe the procedure used in the main text to extract these parameters from the experimental data.

## Bud geometry and energy

We consider a membrane homogeneously covered by identical buds characterized by an opening angle $\theta$ [Fig. 1(a)]. Buds may exhibit morphologies going from non-existent to partial to full buds, and in the following sections we successively study the four subcases illustrated in Fig. 1(b).

### Bare membrane

If clathrin does not bind to the membrane, we have $A_c = 0$ and $\ell = 0$. The membrane is then flat, implying $A_m = A_p$, and thus

$$\frac{f_{\text{bare}}}{\sigma} = 1. \tag{S2}$$

### Shallow partial bud

We consider a bud with an opening angle $\theta \in [0, \pi/2]$, implying $A_c = A_m$. Defining the relative excess area as

$$a = A_m/A_p - 1, \tag{S3}$$

it is easily shown that

$$\cos\theta = \frac{1-a}{1+a}, \qquad \sin\theta = \frac{2\sqrt{a}}{1+a}, \qquad \frac{r_c \ell}{A_p} = \frac{1+a}{\sqrt{a}}. \tag{S4}$$

Defining dimensionless variables

$$\tilde{\mu} = \mu/\sigma \qquad \text{and} \qquad \tilde{\tau} = \tau/(r_c\sigma), \tag{S5}$$

and minimizing the free energy over $a$ we find that for $\tilde{\mu} \leqslant 1$

$$\frac{f}{\sigma} = (1-\tilde{\mu})\frac{(1+a)^2}{1-a}, \qquad \text{with } a \text{ given by} \qquad \frac{1-a}{2a^{3/2}} = \frac{1-\tilde{\mu}}{\tilde{\tau}} \tag{S6}$$

We plot the corresponding opening angle as a function of the dimensionless parameters in Fig. 1(c).

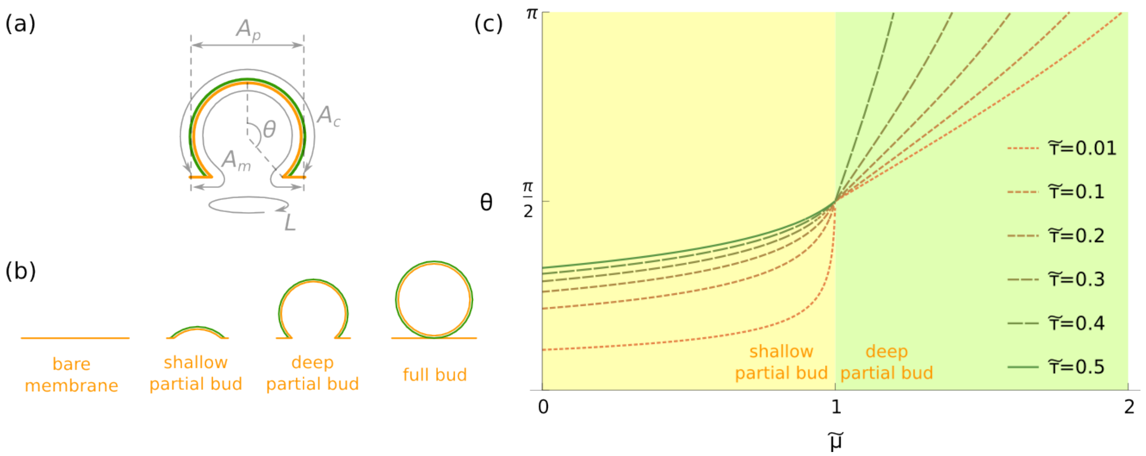


Supplementary Model Figure 1: Individual bud geometry. (a) Parametrization of the problem. The membrane is represented in orange and the clathrin coat in green. (b) Illustration of the four types of coat morphologies consided in Secs. -. (c) Partial bud opening angle as a function of the scaled clathrin adhesion energy $\tilde{\mu}$ for several values of the scaled line tension $\tilde{\tau}$ as given by Eqs. (S4), (S6) for shallow partial buds and Eqs. (S7), (S8) for deep partial buds.

## Deep partial bud

We now consider a bud with an opening angle $\theta \in [\pi/2, \pi]$. As pictured in Fig. 1(a), only the curved part of the membrane is covered by clathrin, implying that some of the membrane is uncovered. As a consequence, the definition of Eq. (S3) now implies

$$\cos\theta = 1 - \sqrt{a}, \qquad \sin\theta = 1 - (1-\sqrt{a})^2, \qquad A_c/A_p = 2\sqrt{a}, \qquad \frac{r_c \ell}{A_p} = 4\sqrt{a} - 2a. \tag{S7}$$

Minimizing with respect to $a$ we find that the resulting membrane free energy has a global minimum for $\theta \in [\pi/2, \pi] \Leftrightarrow a \in [1, 4]$ if and only if $1 \leqslant \tilde{\mu} \leqslant 2$ and $0 \leqslant \tilde{\tau} \leqslant 1 - \tilde{\mu}/2$. We then obtain

$$\frac{f}{\sigma} = 1 - \frac{(\tilde{\mu} - 2\tilde{\tau})^2}{1 - 2\tilde{\tau}}, \qquad \text{with} \qquad a = \left(\frac{\tilde{\mu} - 2\tilde{\tau}}{1 - 2\tilde{\tau}}\right)^2. \tag{S8}$$

We plot the corresponding opening angle as a function of the dimensionless parameters in Fig. 1(c).

## Full bud

A full bud is simply described by the $\theta \to \pi$ limit of the bud discussed in Sec. , implying

$$\frac{f}{\sigma} = 1 + 4(1 - \tilde{\mu}) \qquad \text{and} \qquad a = 4. \tag{S9}$$

# Morphology of the system

Comparing the free energies described in Eqs. (S2), (S6), (S8) and (S9), we plot the stability diagram of the different types of buds in Fig. 2. There, the coexistence lines are given by:

- Bare membrane/full buds: $\tilde{\mu} = 1$
- Bare membrane/shallow buds:

$$\tilde{\tau} = 2\frac{\left[\sqrt{\frac{1}{4(1-\tilde{\mu})^2} + \frac{2}{1-\tilde{\mu}}} - 1 - \frac{1}{2(1-\tilde{\mu})}\right]^{3/2}}{\left[\sqrt{\frac{1}{4(1-\tilde{\mu})^2} + \frac{2}{1-\tilde{\mu}}} - \frac{1}{2(1-\tilde{\mu})}\right]^2}$$

- Shallow/deep buds: $\tilde{\mu} = 1$

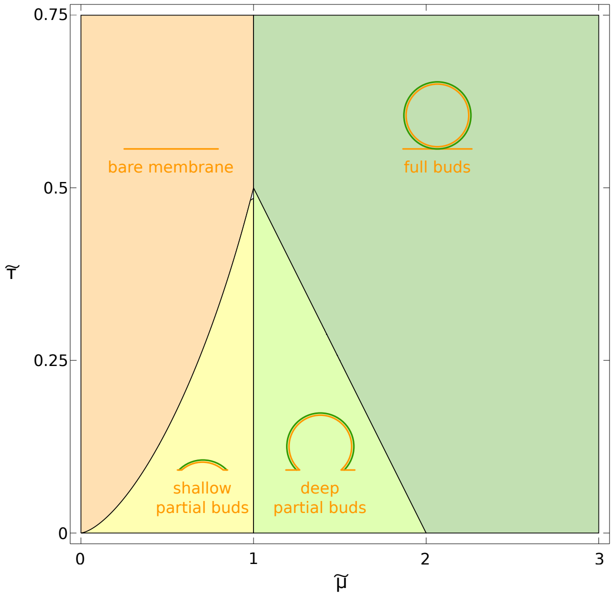


Supplementary Model Figure 2: Bud morphology as a function of the dimensionless clathrin adhesion energy and the line tension.

- deep/full buds: $\tilde{\tau} = 1 - \tilde{\mu}/2$

The stability diagram presented in the main text presents these same regime boundaries in terms of the scaled surface tension $\sigma/(\tau/r_c)$ and adhesion free energy $\mu/(\tau/r_c)$.

# Experimental determination of the clathrin parameters

To determine the two dimensionless parameters $\tilde{\mu}$ and $\tilde{\tau}$ introduced above as well as their dimensional counterparts $\mu$ and $\tau$, we measure two independent quantities for each experiment presented in the main text: the force required to pull a tether from a clathrin-covered giant unilamellar vesicle (GUV) and the change in its membrane surface area upon clathrin polymerization. Here we describe the theoretical procedure used to infer the former from the latter.

The results derived for the infinite flat system case studied above provide us with the membrane's equation of state, which can be used in the more complex experimentally relevant situation pictured in Fig. 3. There, a GUV is held in an aspiration pipette while a membrane tether is pulled from it using optical tweezers. A controlled pressure difference $P_e - P_p$ is maintained between the exterior and the inside of the pipette, allowing a regulation of the membrane tension. To understand this regulation, we first consider a clathrin-less GUV and apply Laplace's law both the the surface of the vesicle and the spherical region at the end of the membrane tongue (the membrane in the aspiration pipette). We find that the membrane has a tension $\sigma_0$ given by

$$P_v - P_e = \frac{2\sigma_0}{R_{v0}}, \quad P_v - P_p = \frac{2\sigma_0}{R_p} \quad \Rightarrow \quad \sigma_0 = \frac{R_{v0}R_p}{2(R_{v0} - R_p)}(P_e - P_p). \tag{S10}$$

A membrane tether of radius $r$ and length $L$ is then extracted from the GUV using optical tweezers. Its free energy is given by $\mathcal{F}_t = 2\pi r L(\sigma_0 + \kappa/2r^2) - F_0 L$, which is composed of a tension, bending energy and pulling force contribution ($\kappa$ is the membrane bending modulus). Minimizing $\mathcal{F}_t$ with respect to $r$ and $L$, we find the tether radius and the force required to extract it:

$$r_0 = \sqrt{\kappa/2\sigma_0}, \quad F_0 = 2\pi\sqrt{2\kappa\sigma_0}. \tag{S11}$$

When clathrin is injected into the setup (in the exterior region), it polymerizes on the vesicle and tether and introduces a modification of the tether force. Denoting by $x$ the difference in membrane tongue length before and after clathrin injection, the system's free energy reads

$$\begin{aligned} \mathcal{F} \quad = \quad & -\pi x R_p^2 P_p + \frac{4\pi R_v^3}{3} P_e + 4\pi R_v^2 f \\ & +\sigma(4\pi R_v^2 A_m/A_p - 4\pi R_{v0}^2 - 2\pi R_p) \\ & +P_v(\pi x R_p^2 - 4\pi R_v^3/3), \end{aligned} \tag{S12}$$

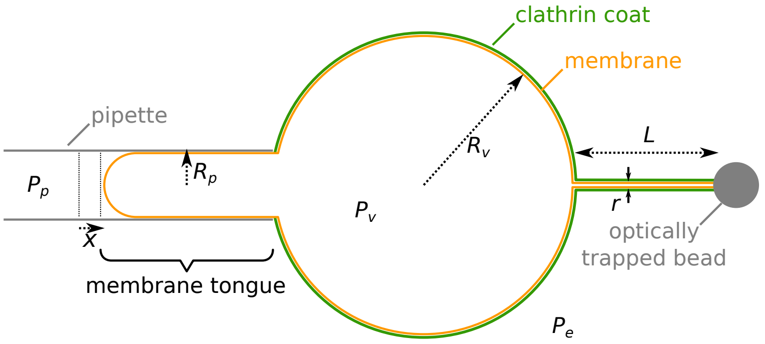


Supplementary Model Figure 3: Schematic of the experimental setup discussed in Sec. and used in the main text.

where the membrane tension $\sigma$ and fluid pressure inside the vesicle $P_v$ are introduced as the Lagrange multipliers respectively enforcing the conservation of membrane area and water volume:

$$4\pi R_v^2 A_m/A_p - 4\pi R_{v0}^2 - 2\pi R_p x = 0 \quad \text{(S13a)}$$
$$4\pi R_v^3/3 - 4\pi R_{v0}^3/3 - \pi R_p^2 x = 0. \quad \text{(S13b)}$$

In this more complicated situation, Laplace's law for the clathrin-coated vesicle and membrane tongue is recovered by minimizing $\mathcal{F}$ with respect to $R_v$ and $R_p$, yielding respectively

$$P_v - P_e = \frac{2f}{R_v}, \quad P_v - P_p = \frac{2\sigma}{R_p}. \quad \text{(S14)}$$

Moreover, the tether free energy is now given by $2\pi r L[\kappa/(2r^2) + f] - FL$, and minimizing with respect to $r$ and $L$ thus yields the tether force

$$F = 2\pi\sqrt{2\kappa f}. \quad \text{(S15)}$$

Combining Eqs. (S10-S11) and (S14-S15), we find

$$\frac{f}{\sigma} = \frac{R_v}{R_p} + \left(\frac{R_v}{R_{v0}} - \frac{R_v}{R_p}\right)\left(\frac{F_0}{F}\right)^2, \quad \text{(S16)}$$

through which the quantity $f/\sigma$ discussed in Sec. can be directly inferred from the experimentally measured tether forces before and after clathrin injection.

We next turn to the change in membrane area following clathrin injection. Using fluorescence microscopy, the GUV radius and tongue length can be monitored throughout the experiment (the area of the membrane tether is negligible). Using the definition of the excess area $a$ introduced in Sec. , we write the membrane area stored in the clathrin-covered part of the GUV as $(1+a)(4\pi R_v^2 - \pi R_p^2)$. We are thus able to assess the amount of membrane area stored in the clathrin buds by writing membrane area conservation, yielding an experimental measure of the excess area

$$a = \frac{4(R_{v0}^2 - R_v^2) + 2R_p x}{4R_v^2 - R_p^2}. \quad \text{(S17)}$$

Finally, the values of the clathrin parameters $\tilde{\mu}$ and $\tilde{\tau}$ are calculated by identifying the expressions Eqs. (S16) and (S17) with those derived in Sec. for the quantities $f/\sigma$ and $a$. In practice, all experimental points are in the shallow bud regime, yielding

$$\tilde{\mu} = 1 - \frac{f}{\sigma}\frac{1-a}{(1+a)^2} \quad \text{(S18a)}$$
$$\tilde{\tau} = = \frac{f}{\sigma}\frac{2a^{3/2}}{(1+a)^2}. \quad \text{(S18b)}$$

The dimensional adhesion free energy and line tension $\mu$ and $\tau$ are then calculated through Eq. (S5), using the experimentally measured value $r_c = 32.5\,\text{nm}$.

Finally, the values of the clathrin parameters $\tilde{\mu}$ and $\tilde{\tau}$ are calculated by identifying the expressions Eqs. (S16) and (S17) with those derived in Sec. S1 for the quantities $f/\sigma$ and $a$. In practice, all experimental points are in the shallow bud regime, yielding

$$\tilde{\mu} = 1 - \frac{f}{\sigma}\frac{1-a}{(1+a)^2} \tag{S18a}$$

$$\tilde{\tau} = = \frac{f}{\sigma}\frac{2a^{3/2}}{(1+a)^2}. \tag{S18b}$$

The dimensional adhesion free energy and line tension $\mu$ and $\tau$ are then calculated through Eq. (S5), using the experimentally measured value $r_c = 32.5\,\mathrm{nm}$.
